# Supplementary material for: The genome sequence of the Scarce Cardinal Beetle, Schizotus pectinicornis (Linnaeus, 1758)
Source: Wellcome Open Res. 2024 Sep 3;9:501. [Version 1] doi: 10.12688/wellcomeopenres.22888.1 (PMC11617827; doi:10.12688/wellcomeopenres.22888.1)
Supplement: Peer review history

# Reviewer report: Reviewer response for version 1

**DOI:** 10.21956/wellcomeopenres.25203.r110789

**Published:** 2024-12-04

**Authors:** Jan Veenstra

**Affiliations:** 1 INCIA, UMR 5287, CNRS, University of Bordeaux, Bordeaux, France

As usual an excellent genome assembly from the Welcome Sanger Institute. I have have only two minor comments. rapid-ensembl.org is being phased out, it may be worth to consider updating the website for this species in the Genome annotation report.

 If possible, update the (article in preparation) citation in Assembly curation.

Are sufficient details of methods and materials provided to allow replication by others?

Yes

Is the rationale for creating the dataset(s) clearly described?

Yes

Are the datasets clearly presented in a useable and accessible format?

Yes

Are the protocols appropriate and is the work technically sound?

Yes

Reviewer Expertise:

Insect neuropeptides

I confirm that I have read this submission and believe that I have an appropriate level of expertise to confirm that it is of an acceptable scientific standard.

# Reviewer report: Reviewer response for version 1

**DOI:** 10.21956/wellcomeopenres.25203.r104093

**Published:** 2024-11-04

**Authors:** Na Ra Shin

**Affiliations:** 1 The University of Memphis, Memphis, Tennessee, USA

This manuscript describes the genome assembly of the Scarce Cardinal beetle ( *Schizotus pectinicornis*), based on PacBio HiFi sequencing, Hi-C data, and RNA sequences from the abdomen. The methods for genome assembly and quality assessment are well described and thorough.

 However, I would appreciate a bit more detail on how the authors addressed potential contamination by symbionts or gut microbes during RNA extraction from the abdomen. Given that *Schizotus pectinicornis* larvae are known to host gut microbiota, though I found no references on adult gut microbiota, it would enhance the rigor of this study if the authors could confirm the absence of contamination in the RNA data extracted from the abdomen. 

 Other than that, I have no major objections to the data presented in this manuscript.

Are sufficient details of methods and materials provided to allow replication by others?

Partly

Is the rationale for creating the dataset(s) clearly described?

Yes

Are the datasets clearly presented in a useable and accessible format?

Yes

Are the protocols appropriate and is the work technically sound?

Yes

Reviewer Expertise:

Beetles evolution, Systematic, Metabolic evolution

I confirm that I have read this submission and believe that I have an appropriate level of expertise to confirm that it is of an acceptable scientific standard.
